# Cardiac tamponade due to influenza B infection in a young immunocompetent female: A case report and review of literature

**DOI:** 10.5339/qmj.2025.26

**Published:** 2025-03-17

**Authors:** Sreethish Sasi, Fatma Ben Abid, Mohammed Altayeb Alamin, Javed Iqbal, Muna Al-Maslamani

**Affiliations:** ^1^Infectious Diseases Division, Department of Medicine, Communicable Diseases Center, Hamad Medical Corporation, Doha, Qatar; ^2^Department of Nursing, Communicable Diseases Center, Hamad Medical Corporation, Doha, Qatar *Correspondence: Sreethish Sasi. Email: Ssasi7@hamad.qa

**Keywords:** Influenza, tamponade, case report

## Abstract

**Background:**

Influenza infections are recognized globally for their respiratory manifestations, but are less commonly associated with severe cardiovascular complications such as cardiac tamponade. The relationship between influenza infections, particularly influenza B, and cardiac complications such as myocarditis, pericarditis, and cardiac tamponade remains underexplored, particularly in immunocompetent individuals.

**Case summary:**

We report the case of a 22-year-old immunocompetent female who presented to the emergency department with acute shortness of breath, fatigue, and dizziness due to symptoms suggestive of an influenza-like illness. Laboratory and imaging findings revealed a large circumferential pericardial effusion suggestive of cardiac tamponade. Subsequent investigations confirmed influenza B infection. The patient was managed with pericardiocentesis, oseltamivir, nonsteroidal anti-inflammatory drugs, colchicine, and supportive care, resulting in complete recovery. This case highlights the significance of considering influenza as a potential cause of acute cardiac complications and the importance of early diagnostic and therapeutic interventions to prevent morbidity and mortality.

**Discussion:**

The occurrence of cardiac tamponade secondary to influenza B infection in a young, immunocompetent female highlights the critical need to educate healthcare providers about the potential cardiovascular complications of influenza. The mechanisms underlying influenza-associated cardiac involvement may include direct viral invasion, systemic inflammation, and immune-mediated responses.

**Conclusion:**

This case contributes to the limited but growing body of literature on influenza-induced cardiac complications and highlights the importance of timely antiviral therapy alongside traditional management strategies for cardiac tamponade. Further research is needed to elucidate the pathophysiology of influenza-related cardiac complications and to provide guidelines for the management of such cases.

## Introduction

Influenza infections represent a significant global health concern, causing a variety of symptoms ranging from mild, self-limiting infections to severe respiratory failure and death.^1^ The causes of these complications are poorly understood, with both host- and virus-specific factors being involved.^1–3^ Severe pneumonia and respiratory failure often occur after influenza infections.^1,2^ The ability of influenza viruses to spread to distant organs and cause tissue damage outside the lungs is also not fully understood.^1,2^ Cardiac involvement in influenza infections is rare, with influenza myocarditis typically presenting as a mild, self-limiting disease.^2^ Fulminant shock is rarely reported, and there are only a few case reports of cardiac complications due to influenza B infections in adults.^1–6^ Influenza viruses affect the upper and lower respiratory tracts, leading to increased morbidity and mortality. Although cardiovascular complications are rare, studies suggest an association between influenza and increased cardiovascular mortality, particularly during the flu season.^3,4,7^ Primary cardiac pathologies such as myocarditis, pericarditis, pericardial effusion, and cardiac tamponade caused by influenza B are not well defined.^8^ Understanding the clinical characteristics, diagnostic approaches, treatment strategies, complications, and outcomes of patients with influenza-related myopericarditis or isolated pericarditis is crucial due to the annual public health burden of influenza. Cardiac tamponade, a life-threatening condition, can occur as a complication of influenza B infection,^1–9^ highlighting the need for comprehensive data on demographic and clinical characteristics as well as management strategies to reduce mortality in high-risk patients. Hamad Medical Corporation (HMC) is the primary healthcare provider for secondary and tertiary care for almost 3 million people in the State of Qatar (10). It has 14 general and specialty hospitals with a bed capacity of nearly 2,500, multiple specialized outpatient services, and national electronic medical records (10). The publication of this case report was approved by the IRB (Institutional Review Board), Medical Research Centre (MRC) of HMC (approval no. MRC-04-24-160). Written and informed consent was obtained from the patient for the publication of her case information and images.

## Case Presentation

**Figure 1.**
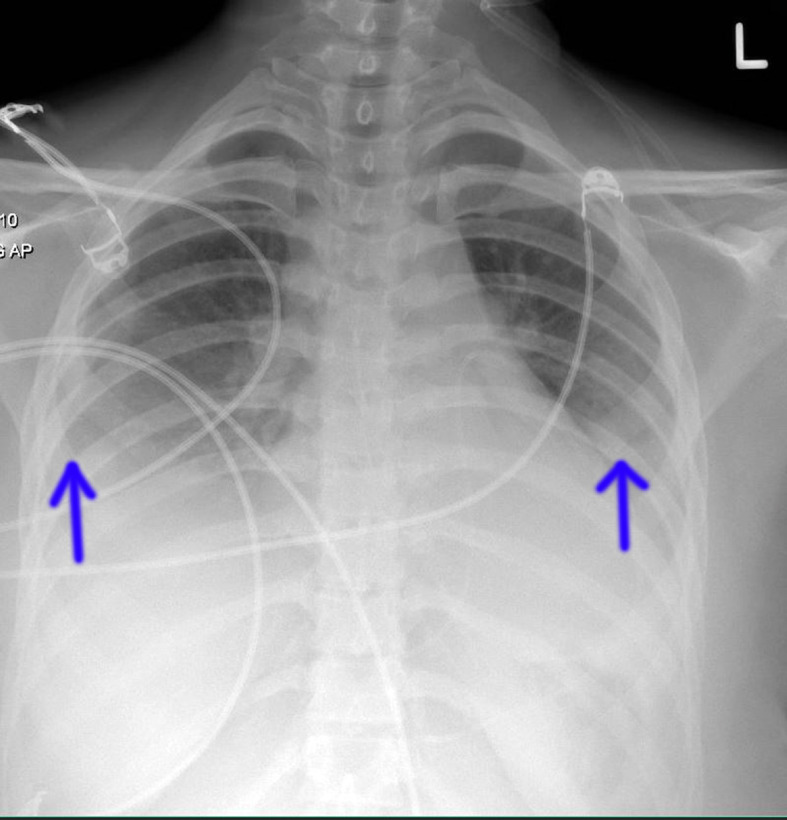
Chest X-ray at the time of initial presentation showing bilateral obliteration of the costophrenic angles, more pronounced on the right side, and haziness in both lower lung fields (blue arrows).

**Figure 2.**
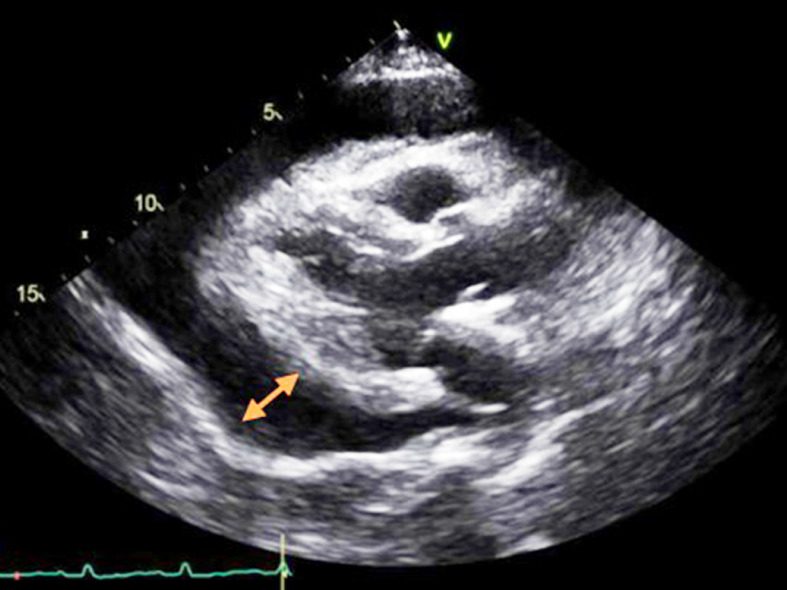
Transthoracic echocardiogram showing large pericardial effusion (orange arrow) with impending cardiac tamponade.

A 22-year-old Qatari woman presented to the emergency department of Hamad General Hospital, Doha, Qatar, in November 2023 with acute shortness of breath, fatigue, and dizziness after suffering from fever, vomiting, and watery diarrhea for three days. She had no relevant medical, surgical, family, or psychosocial history. She was found to have respiratory distress, with a respiratory rate of 28 breaths per minute, room air oxygen saturation of 92%, a heart rate of 110 beats per minute, and a blood pressure of 100/70 mmHg. Laboratory investigations revealed an elevated white blood cell count of 13 × 10^9^ per liter, a C-reactive protein level of 2.7 milligrams per liter, and a procalcitonin level within normal limits. A chest X-ray revealed bilateral obliteration of costophrenic angles, more pronounced on the right side, and haziness in both lower lung fields (Figure 1). Transthoracic echocardiography (TTE) showed a large collection anterior to the right ventricular free wall, a variation in transmitral valve flow of more than 25%, a non-collapsing inferior vena cava (right atrial pressure between 5 and 10 mmHg), a systolic collapse of the right atrium, and right ventricular early diastolic collapse suggestive of cardiac tamponade (Figure 2). The left ventricular ejection fraction was 49%. Pericardiocentesis was performed and 400 milliliters of pericardial fluid was aspirated. It was a straw-colored fluid with a clear appearance. The white blood cell count in the fluid was increased to 211 cells per microliter, predominantly consisting of monocytes (90%) and a small percentage of lymphocytes (8%). Viral panel analysis of the pericardial fluid revealed negative results, and autoimmune workup, including serological tests, was unremarkable. A nasopharyngeal and throat swab was positive for influenza B, while all other respiratory pathogen polymerase chain reaction (PCR) tests were negative. The patient was promptly started on oseltamivir, nonsteroidal anti-inflammatory drugs (NSAIDs), colchicine, and supportive care. Her condition improved, and she was discharged after a week of hospitalization. Viral myocarditis was considered as part of the differential diagnosis, but given the clinical improvement after pericardiocentesis and medical therapy, further evaluation with cardiac MRI was not pursued. Serial testing of cardiac troponin was performed and was within normal limits. A clinical follow-up was performed one week after hospital discharge, during which the patient was asymptomatic and a TTE showed an EF (Ejection Fraction) of 55% without pericardial effusion.

## Discussion

The case presented highlights the importance of considering influenza infections as a potential cause of acute cardiac complications, particularly in young adults with respiratory symptoms. Influenza viruses are well known for their respiratory manifestations. However, their association with cardiac complications, although rare, can significantly impact patient outcomes and requires prompt recognition and management.

**Figure 3.**
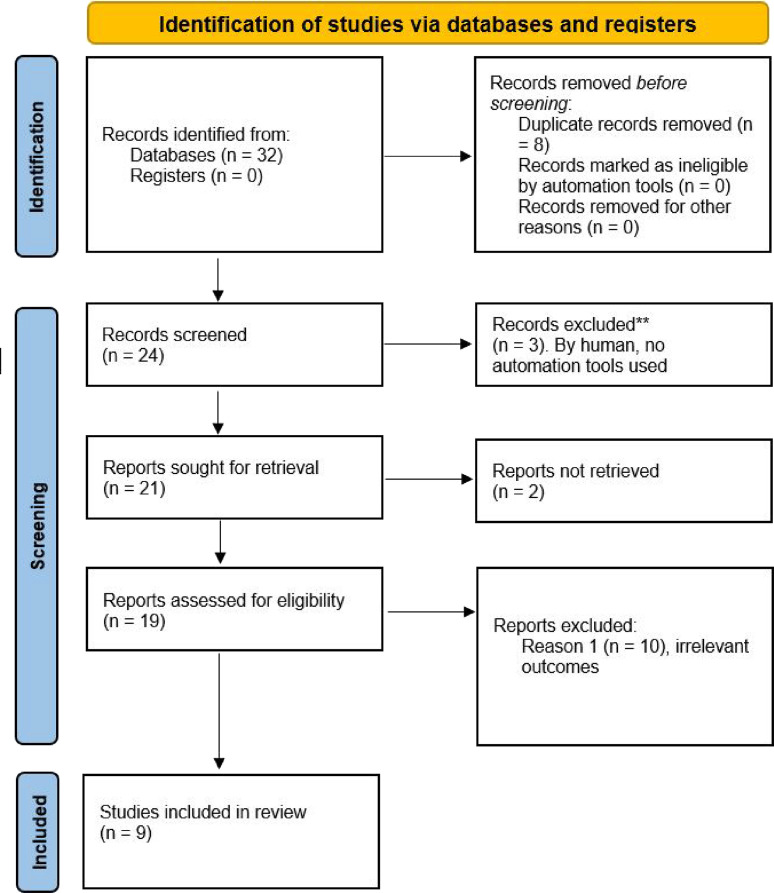
Representation of the literature search using a PRISMA (Preferred Reporting Items for Systematic Reviews and Meta-Analyses) flow diagram.

Pericardial effusion and cardiac tamponade, as observed in this case, are rare complications of influenza infections, especially in the absence of underlying cardiovascular disease. A literature review was conducted using a systematic approach, focusing on studies of cardiac tamponade associated with influenza B infection. We searched PubMed, Scopus, and Google Scholar for articles published between 2000 and the present, using keywords such as “Influenza B”, “cardiac tamponade”, and “viral myocarditis”. We included peer-reviewed case reports and reviews that specifically addressed cardiac complications associated with influenza B in immunocompetent individuals. Articles were screened for relevance, and data on patient demographics, clinical presentation, diagnosis, treatment, and outcomes were extracted and analyzed. The PRISMA (Preferred Reporting Items for Systematic Reviews and Meta-Analyses) flow diagram for the literature search is shown in Figure 3. The findings were synthesized to identify patterns and inform the discussion in the manuscript. While the exact mechanism of influenza-induced cardiac involvement remains unclear, several hypotheses have been proposed.^2–10^ Direct viral invasion of cardiac tissue leading to myocarditis, systemic inflammation triggered by the viral infection, and immune-mediated responses have been identified as potential pathways leading to pericardial effusion and tamponade.^3,8,9^ TTE played a pivotal role in the diagnosis and management of the patient by revealing a large circumferential pericardial effusion with signs of cardiac tamponade. Prompt pericardiocentesis, taking into account echocardiographic findings, was crucial for relieving cardiac compression and preventing further hemodynamic compromise. Detection of influenza B virus in respiratory samples highlights the importance of considering viral etiologies in patients with acute respiratory symptoms, especially during the influenza season. Timely initiation of antiviral therapy with oseltamivir likely contributed to the patient's favorable outcome by reducing viral replication and limiting disease progression. Although pericardial fluid analysis did not reveal viral presence, negative results do not rule out influenza-induced pericarditis because viral shedding in the pericardial fluid may occur intermittently or at low levels. Additionally, the absence of autoimmune markers suggests that the pericardial effusion was likely a consequence of the viral infection rather than an autoimmune-mediated process. A comparative analysis of our case with existing cases of influenza related cardiac tamponade in the literature is summarized in Table 1. A statistical analysis of the cases presented shows an unequal distribution between influenza A and influenza B infections (95:39). The outcomes vary, with a higher recovery rate of 75% compared to a fatality rate of 25%. Pericardiocentesis was performed in all cases, indicating its widespread use as a therapeutic intervention. The most commonly administered medical treatment was oseltamivir, which was used in all cases. Broad-spectrum antibiotics were also used frequently,^1,5,6^ while other treatments such as NSAIDs, corticosteroids, and colchicine^1,2,8^ were used less frequently. This analysis underscores the importance of tailored medical interventions in managing influenza-related complications, highlighting the variability in treatment approaches across different cases. Prospective studies investigating the long-term cardiovascular sequelae of influenza infections are needed to better understand the implications of influenza on cardiac health beyond the acute phase of the illness.

The case highlights the importance of TTE in diagnosing large circumferential pericardial effusion with signs of cardiac tamponade, as recommended by the European Society of Cardiology (ESC) guidelines.^11,12^ TTE is the first-line imaging modality for diagnosing pericardial effusion and cardiac tamponade, and the guidelines recommend immediate management with pericardiocentesis to relieve pressure from cardiac tamponade.^11,12^ Management of pericarditis, including antiviral therapy with oseltamivir, NSAIDs, and colchicine, is in accordance with the ESC guidelines for the treatment of viral pericardial disease. The manuscript's approach to providing supportive care and detailed follow-up, demonstrating improvement and resolution of pericardial effusion, is consistent with comprehensive care strategies recommended by the ESC, particularly in the acute phase of the disease.^11,12^

## Conclusion

This case report highlights the importance of considering influenza B as a potential cause of severe cardiac complications, even in young and otherwise healthy individuals. The successful management of cardiac tamponade in this patient highlights the critical role of timely diagnosis and intervention, particularly the use of TTE and pericardiocentesis. The patient's complete recovery following antiviral therapy, NSAIDs, and colchicine further emphasizes the effectiveness of early and appropriate treatment strategies. As influenza can lead to life-threatening cardiac conditions, healthcare providers should maintain a high index of suspicion during the influenza season. This case adds to the growing body of literature on influenza-induced cardiac complications and serves as a reminder that vigilance and prompt treatment are required in such cases.

### List of abbreviations

[Table tbl2]

### Competing interests

The authors have no conflicts of interest to declare.

### Authors' contributions

**SS** conceptualized the study, drafted and revised the manuscript, and gave final approval. **FBA**, **MAA**, and **JI** conducted literature reviews and analyses, critically revised the manuscript, and approved the final version. **MAM** reviewed and interpreted imaging and laboratory findings, contributed to drafting and revising the manuscript, and gave final approval for publication.

## Figures and Tables

**Table 1:** Summary of existing literature on cardiac tamponade in patients with influenza infection.

**Sl. no.**	**Author**	**Year**	**Type of article**	**Journal**	**No. of cases**	**Age (years)**	**Sex**	**Presenting symptoms**	**Diagnosis based on sample**	**Influenza type**	**Outcomes**	**Mortality**	**Pericardiocentesis**	**Medical treatment administered**

1.	Dominick Roto et al.^1^	2018	Case report	Case Reports in Critical Care	1	57	Female	Cardiac tamponade, refractory shock, death	Autopsy, postmortem cardiac tissue	B	Fatal	Yes	Yes	IV fluids, high-dose vasopressors, broad-spectrum antibiotics, stress-dose steroids

2.	Cristian Dumitrescu et al.^2^	2019	Abstract of case report	Chest	1	47	Male	Shortness of breath, chest pressure, elevated jugular venous pulse, distant heart sounds	PCR of nasopharyngeal swab	B	Recovered	No	Yes	6-week course of colchicine twice daily and ibuprofen 3 times a day for 6 weeks

3.	Yadav Pandey et al.^3^	2019	Case report	Clinical Medicine	1	57	Male	Chest pain, fever, muscle aches	Rapid influenza diagnostic test	A	Recovered	No	Yes	Oseltamivir for 5 days

4.	Rupak Desai et al.^7^	2020	Systematic review	Circulation	28 (19 adults, 9 pediatrics)	4–69	13 males, 15 females	Dyspnea, chest pain	PCR of nasopharyngeal swab	A – 78.5%, B –10.7%	Recovered–82.1%, died – 14.2%	Yes	25 (89.2%) patients	8 (28.5%) with oseltamivir, 2 (7.1%) with oseltamivir followed by peramivir, 1 (3.5%) with zanamivir, 1 (3.5%) with zanamivir followed by peramivir

5.	Arfaras-Melainis et al.^4^	2020	Case report	Cureus	1	32	Female	Exertional dyspnea, pericardial effusion, cardiac tamponade	PCR of nasopharyngeal swab	B	Recovered	No	Yes	Oseltamivir for 5 days

6.	Junaid Mir et al.^5^	2021	Case report	Chest	1	31	Male	Dyspnea, cough, tachypnea, hypotension, cardiac arrest	PCR of nasopharyngeal swab	B	Comfort care	No	Yes	He was given a bolus of normal saline and started on broad-spectrum antibiotics

7.	Schroff P et al.6	2021	Case report	Cureus	1	29	Female	Chest pain, fever, myalgia, pericardial effusion	PCR of nasopharyngeal swab	B	Recovered	No	Yes	Five-day course of oseltamivir and a seven-day course of azithromycin

8.	Radovanovic, M. et al.^8^	2022	Literature review	J. Clin. Med.	75 (55 adults and 20 pediatrics)	32.3 ± 18.8	31 males, 44 females	Influenza pericarditis and myopericarditis	Not specified	25 B, 50 A	63 recovered, 1 awaiting transplant, 11 deceased	11 – 9 influenza A, 2 influenza B	Yes	Antivirals NSAIDs Corticosteroids Colchicine and intra-venous immunoglobulin (IVIG) Circulatory support Mechanical interventions Pericardiocentesis (37.3%) of cases Pericardiectomy (5.3%) Pericardial window 4%

9.	Desai et al.^9^	2023	Review	SN Comprehensive Clinical Medicine	25 (19 adults, 6 pediatrics)	Adults (47.6 ± 15.12) and pediatrics (10.1 ± 4.5)	15 (60%) females and10 (40%) males	Fever, weakness, dyspnea, cough, chest pain	PCR of nasopharyngeal swab	19 A, 6 B	22 recovered, 3 died	Yes (3 patients)	Yes	Varied from patient to patient

10.	This Case	2023	Case report		1	22	Female	Acute respiratory distress	PCR of nasopharyngeal swab	B	Recovered	No	Yes	Oseltamivir, NSAIDs, colchicine, and supportive care


**Table tbl2:** 

EF	Ejection Fraction

NSAIDs	Nonsteroidal Anti-Inflammatory Drugs

PCR	Polymerase Chain Reaction

TTE	Transthoracic Echocardiography

IVIG	Intra-venous immunoglobulin

